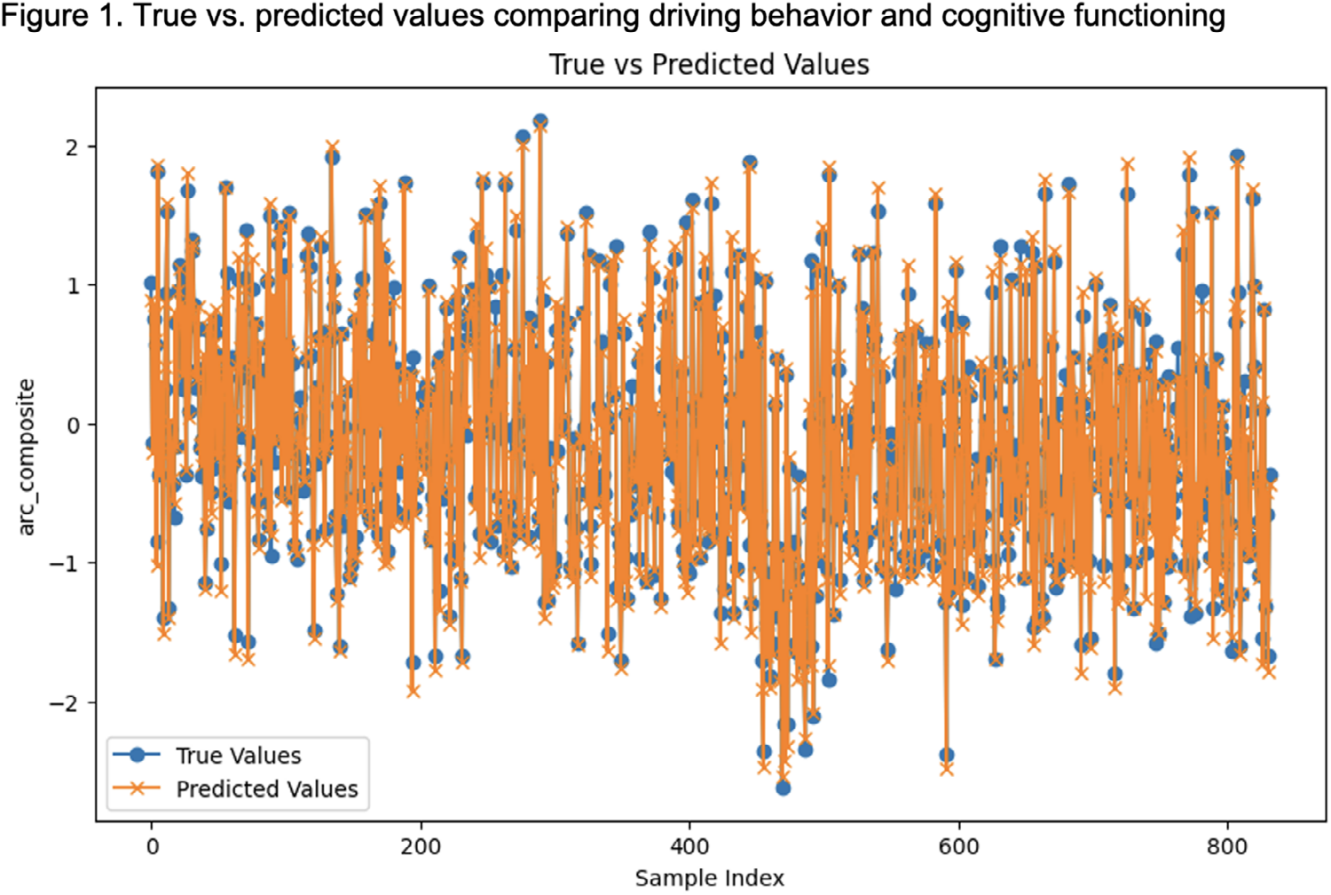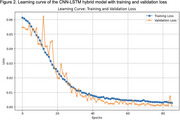# Revolutionizing predictive analytics: A hybrid CNN‐LSTM approach to predicting cognitive status using driving behavior analysis

**DOI:** 10.1002/alz70856_102164

**Published:** 2025-12-25

**Authors:** Noor Al‐Hammadi, Ganesh M. Babulal, The DRIVES Project

**Affiliations:** ^1^ Washington University School of Medicine, SAINT LOUIS, MO, USA; ^2^ Washington University School of Medicine, Saint Louis, MO, USA; ^3^ University of Johannesburg, Johannesburg, Gauteng Province, South Africa

## Abstract

**Background:**

Measuring cognition at short intervals is increasingly common to capture substantial fluctuations over time. However, it remains unknown if daily variations in cognitive performance affect real‐world behaviors. Analyzing driving behavior data is challenging due to nonlinear relationships and temporal dependencies, which traditional linear models cannot effectively capture. This study assesses a hybrid Convolutional Neural Network‐Long Short‐Term Memory (CNN‐LSTM) model to predict cognitive functioning from driving metrics. The model utilizes convolutional layers for feature extraction and LSTM layers for sequential analysis, offering a novel method for understanding complex driving patterns.

**Method:**

Data were sourced using brief cognitive tests at random intervals four times daily for months via smartphone, while driving behavior metrics were collected simultaneously. Feature engineering developed variables like Distance Ratio and Short Trip Ratio, along with normalization and Gaussian noise augmentation for robustness. The dataset was transformed into sequences with a sliding window approach for temporal coherence. A hybrid CNN‐LSTM model was created, featuring Conv1D layers for spatial feature extraction and stacked LSTM layers for temporal dependencies. The model was trained with the Adam optimizer, Huber loss, and early stopping to prevent overfitting. Evaluation metrics included Mean Absolute Error (MAE) and R‐squared (R^2^).

**Result:**

Feature correlation analysis revealed weak but statistically significant relationships for Distance Ratio (negative) and Short Trip Ratio (positive) with the target variable. Feature importance analysis identified Distance Ratio as the most predictive metric. The CNN‐LSTM model demonstrated exceptional performance, achieving an R^2^ of 0.9856 and an MAE of 0.0321. Predictions closely aligned with actual values, indicating strong generalization. Learning curves highlighted consistent improvements, confirming the model's robustness and avoiding overfitting.

**Conclusion:**

This study underscores the efficacy of hybrid CNN‐LSTM models for capturing nonlinear and temporal dependencies in cognitive functioning and driving behavior data. Feature engineering, combined with advanced modeling techniques, enhances predictive capabilities and reveals nuanced relationships in the data. Future research could explore alternative architectures, such as attention mechanisms, and integrate external factors like weather or traffic conditions to improve predictive accuracy further. This innovative approach demonstrates significant potential for broader applications in predictive analytics involving sequential data.